# Metabolite analysis distinguishes between mice with epidermolysis bullosa acquisita and healthy mice

**DOI:** 10.1186/1750-1172-8-93

**Published:** 2013-06-26

**Authors:** Sarah Schönig, Andreas Recke, Misa Hirose, Ralf J Ludwig, Karsten Seeger

**Affiliations:** 1 Excellence Cluster Inflammation at Interfaces, Schleswig-Holstein, Germany; 2 Department of Chemistry, University of Lübeck, Ratzeburger Allee 160, Lübeck 23562, Germany; 3 Department of Dermatology, University of Lübeck, Ratzeburger Allee 160, Lübeck 23562, Germany

**Keywords:** Metabolism, Epidermolysis bullosa acquisita, ^1^H-NMR, Type VII collagen

## Abstract

**Background:**

Epidermolysis bullosa acquisita (EBA) is a rare skin blistering disease with a prevalence of 0.2/ million people. EBA is characterized by autoantibodies against type VII collagen. Type VII collagen builds anchoring fibrils that are essential for the dermal-epidermal junction. The pathogenic relevance of antibodies against type VII collagen subdomains has been demonstrated both *in vitro* and *in vivo*. Despite the multitude of clinical and immunological data, no information on metabolic changes exists.

**Methods:**

We used an animal model of EBA to obtain insights into metabolomic changes during EBA. Sera from mice with immunization-induced EBA and control mice were obtained and metabolites were isolated by filtration. Proton nuclear magnetic resonance (NMR) spectra were recorded and analyzed by principal component analysis (PCA), partial least squares discrimination analysis (PLS-DA) and random forest.

**Results:**

The metabolic pattern of immunized mice and control mice could be clearly distinguished with PCA and PLS-DA. Metabolites that contribute to the discrimination could be identified via random forest. The observed changes in the metabolic pattern of EBA sera, i.e. increased levels of amino acid, point toward an increased energy demand in EBA.

**Conclusions:**

Knowledge about metabolic changes due to EBA could help in future to assess the disease status during treatment. Confirming the metabolic changes in patients needs probably large cohorts.

## Background

Epidermolysis bullosa acquisita (EBA) is a rare chronic mucocutaneous autoimmune skin blistering disease with a prevalence of approximately 0.2 per million people [1]. It was first described at the end of the 19th century [2] as a descriptive clinical diagnosis for patients with adult onset and features resembling those of hereditary dystrophic epidermolysis bullosa. Almost 70 years later EBA was distinguished from other bullous diseases on the basis of distinctive clinical and histological features, implementing the first diagnostic criteria for the disease [3]. In relative rapid succession, type VII collagen (COL7) was identified as the autoantigen of EBA [4], and the pathogenic relevance of anti-COL7 antibodies has been demonstrated both in vitro, as well as in vivo [5,6]. In addition, development of animal models has greatly improved our understanding of EBA pathogenesis. This includes unraveling of the genetic basis of disease development [7,8], as well as identification of potential novel therapeutic targets; e.g. HSP90 or PI3Kβ [9,10].

Despite these detailed insights into the pathogenesis of EBA, our current understanding of pathophysiological pathways in EBA is far from complete. Identification of metabolites in different bodily fluids by NMR (nuclear magnetic resonance) spectroscopy has become a valuable tool to analyze metabolic changes during disease [11]. Alteration in bodily fluids can be seen promptly after a change in condition [12] and the amount of metabolites is manageable. Various autoimmune diseases have been characterized by such a metabolomic approach [13], the inflammatory bowel diseases belonging to the most extensively investigated. Metabolite analysis of patients with an inflammatory bowel disease like Crohn’s disease or ulcerative colitis allowed discrimination of diseased persons and a healthy control group [14,15]. The urinary and plasma metabolite profile of an interleukin 10 deficient mouse model (IL-10 −/−) of Crohn’s disease [16,17] revealed alterations in metabolic pathways that could be related to intestinal inflammation.

In another inflammatory disease, rheumatoid arthritis, metabolites involved in nucleic acid, amino acid and fatty acid metabolism differed between rheumatoid and control mice, thus representing a metabolic profile associated with that disease [18]. In patients with rheumatoid arthritis, metabolic profiles could be related to disease severity [19].

Furthermore, non-inflammatory diseases have also been characterized by metabolic profiling via NMR, like the autosomal dominant polycystic kidney disease (ADPKD) and diabetes. Urinary profiles showed differences between patients with ADPKD, patients with other kidney diseases and individuals with normal kidney function, due to increased excretion of proteins and methanol in ADPKD [20]. The metabolic pattern of streptozotocin-induced diabetic rats differs from that of control rats as a result of enhanced levels of triglycerides, fatty acids and acetoacetate in diabetic rats [21].

Advantages of using NMR spectroscopy for metabolic profiling are facile sample preparation, the investigation of a wide range of metabolites in one spectrum and the possibility to recover samples after measurement. Moreover, NMR can be used as a non-targeted method [22] enabling the identification of changes in metabolites that were not under previous scrutiny. NMR spectra are too complex for straightforward analysis, therefore interpretation of the data demands simplification. This is achieved through statistical methods like principal component analysis (PCA) [23] and PLS-DA [24].

Until now only few studies examined the influence of skin related diseases on the metabolite changes in blood e.g. for peanut allergy [25], leprosy [26] or systemic lupus erythematosus [27]. We here present a metabolic approach to identify changes in serum that occur during an autoimmune skin blistering disease. Sera of a mouse model of EBA were analyzed by proton NMR spectroscopy and compared to sera of control animals to ascertain if their metabolic profiles differ.

## Methods

### Induction of experimental EBA

SJL mice (Charles River, Sulzfeld, Germany) aged 8–10 weeks were used for the experiments. Mice were held at specific pathogen free conditions, and fed standard mouse chow and acidified drinking water *ad libitum*. Experimental EBA was induced by immunization with a GST-tagged immunodominant protein located within murine COL7 (GST-mCOL7c) as described [28]. Control mice were immunized with GST emulsified in adjuvant TiterMax® (HiSS Diagnostic GmbH, Freiburg, Germany).

As induction of experimental EBA requires the COL7 antigen and an adjuvant, mice immunized with GST + adjuvant have been selected as a control. Mice treated solely with TiterMax® represent not an ideal control group since the immune response stimulated by the antigen is missing.

Mice were anesthetized with carbon dioxide and blood samples were collected by heart puncture nine weeks after immunization in study A (14 EBA and 15 control mice) and thirteen weeks in study B (15 mice for each condition). At these time points extend of experimental EBA was assessed by determination of the body surface area affected by EBA skin lesions. Animal experiments were approved by local authorities of the Animal Care and Use Committee (Kiel, Germany) and performed by certified personnel.

### Preparation of NMR samples

After blood collection all steps until sample preparation were performed at 4°C. Blood samples were spun down directly after extraction. The supernatant was ultrafiltrated using Vivaspin 500 filters (Sartorius Stedim) with a 3 kD MW cut-off to separate proteins and cell components from the metabolites of interest [18]. Filters were prewashed four times with water to remove preservatives. Sodium phosphate buffer, D_2_O and 3-(trimethylsilyl) propionic acid-*d*_4_ sodium salt (TSP-*d*_4_) were added to the filtrate to obtain final concentrations of 0.1 M sodium phosphate buffer pH 7, 10 to 50 μM TSP-*d*_4_ and 10% D_2_O and a final volume of 180 μl. Samples were stored at −80°C.

### Data acquisition and processing

Samples were thawed and transferred to a 3 mm NMR tube immediately before spectrum acquisition. ^1^H NMR spectra were recorded at 298 K with 512 transients on a Bruker DRX 500 spectrometer equipped with a TCI cryoprobe. The standard noesypr1d pulse sequence was used with parameters as described in [29]. Spectra were processed with Bruker Topspin 2.1. Phasing, baseline correction and referencing were performed manually.

Bucketing of the spectra was done with Amix 3.9.2 (Bruker) with a bucket width of 0.02 ppm (rectangular bucketing). The spectral regions of the water signal, glucose and lactate (6.3 to 3.2 ppm and 1.41 to 1.22 ppm) have been excluded from bucketing. Each bucket was integrated and scaled to total spectral intensity. Values of integrals were exported and transferred to Gnu R for statistical analysis.

Metabolites were identified via reference spectra recorded at 298 K. Sample composition for reference spectra was 20 mM of reference compound, 1 mM TSP-*d*_4_, 0.1 M sodium phosphate buffer pH 7, 10% D_2_O and 0.02% sodium azide.

### Statistics

Gnu R open-source statistical software (version 2.14) was used for statistical analyses and graphical representation of data. For principal components analysis (PCA), a robust method (“PcaHubert”) was used provided by R package “rr-cov” (version 1.3-01, Todorov and Filzmoser, 2009). Outlier spectra, identified by this robust PCA method, were excluded from further analysis, including partial least squares discrimination analysis (PLS-DA). For PLS-DA, the method provided by R package “pls” (version 2.3-0) was used in combination with a jackknife testing method for relevance of single buckets in complete spectra. For random forest analyses, package “randomForest” was was used. For variable selection, the accuracy, i.e. the fraction of correctly classified “out-of-bag” samples, was taken as importance criterion.

## Results and discussion

Although a multitude of data on clinical and immunological aspects of EBA is available, the impact of EBA on metabolism has not been investigated yet. The knowledge of metabolic changes during EBA can give insight into the onset of this disease and points to possible new targets in therapy. In this study the metabolite pattern of experimental EBA was analyzed by proton NMR spectroscopy and compared to sera of control animals.

### Induction of experimental EBA

As described previously [30,31], 100% (study A) and 93% (study B) of SJL mice immunized with an immunodominant fragment of murine COL7 (mCOL7) developed experimental EBA within the observation period of 9 or 13 weeks respectively. In study A, serum was obtained for analysis 9 weeks after immunization. At this time point, mean disease severity, expressed as percentage of body surface area affected by EBA lesions, reached a score of 5.7 ± 1.3. Disease severity ranged from 0.8 (minimum) to a maximum of 15.1. In study B, that was concluded 13 weeks after immunization average disease severity amounted to 6.8±1.7 (range: 0 to 21.4). A box plot of the disease score for both studies is shown in Additional file 1: Figure S1.

### COL7-immunized and control mice differ in their metabolic profile

**Figure 1:**
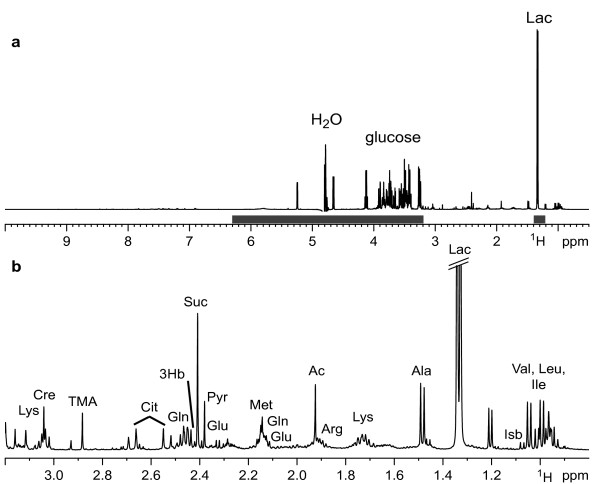
**500 MHz proton spectrum of protein free serum from an EBA mouse.** The spectrum is dominated by glucose and lactate (Lac) as the two metabolites with the highest concentration in the sample (**a**). The solid bars indicate the regions excluded from bucketing. (**b**) Expansion of the aliphatic region. Labels indicate identified metabolites. Amino acids are labeled according to their three letter code; Ac, acetate; Cit, citrate; Cre, creatine; 3Hb, 3-hydroxybutyrate; Isb, isobutyrate; Pyr, pyruvate; Suc, succinate; TMA, trimethylamine.

**Figure 2:**
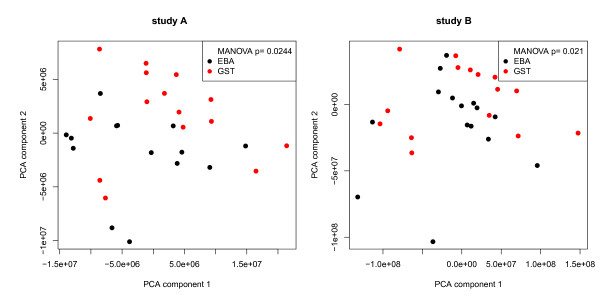
**Principal component analysis (PCA).** Robust PCA allows discrimination of mCOL7-immunized mice and control groups in studies **A** and **B** along the first two PCA components. Dots represent results from individual mice.

**Figure 3:**
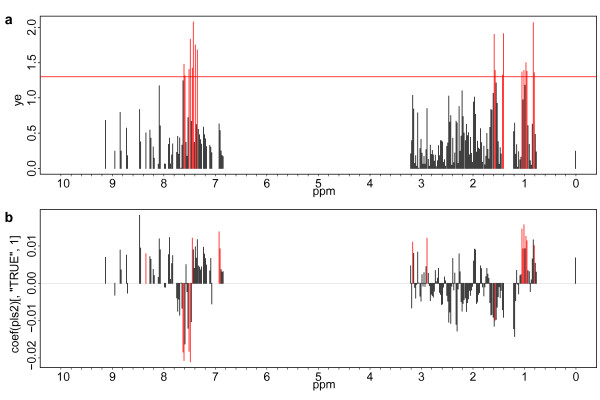
**Identification of significant buckets for the discrimination of EBA and control sera.** (**a**) Bar plot showing the p values from jackknife testing for PLS-DA (study A). Buckets with a p value below 0.05 by jacknife t test for PLS are displayed in red. (**b**) Bar plot showing coefficients from PLS-DA. Important buckets are colored red. Positive column orientation represents a higher peak intensity in this bucket as found in the control state, negative column orientation correlates with a lower peak intensity indicating EBA.

In two studies we obtained ^1^H NMR spectra from EBA mice immunized with mCOL7 (study A 14 mice, study B 15 mice) and control mice mock-immunized with GST protein (15 mice each). For all immunizations TiterMax® was used as adjuvant. A representative NMR spectrum of a mCOL7-immunized mice from study B is shown in Figure 1. Due to their complexity NMR spectra were bucketed and subjected to statistical analysis. We used a filter to suppress noise lower than 5% of the overall spectrum intensity. The statistical analysis comprised PCA, PLS-DA as well as random forest. The results from the different algorithms are similar but not identical, as was also seen in other studies using different statistical approaches [32]. For both studies discrimination between EBA and control mice can be seen in a combination of component 1 and 2 (study A: p = 0.0244; study B: p = 0.021; MANOVA; Figure 2, see also Additional file 1: Figures S2 and S3). With PLS-DA buckets significant for discrimination were identified. In study A significant buckets had peaks of the following metabolites: histidine, isoleucine, leucine, phenylalanine, tyrosine and valine (Table 1, Figure 3). For study B PLS-DA found significant buckets that could be assigned to lysine and tryptophane (Table 1). The data was also evaluated with random forest and the 30 most important buckets for the discrimination between EBA and control mice were considered. With this statistical approach more metabolites/buckets have been found to be significant for discrimination of EBA and control mice (Table 1). In both studies the amino acids isoleucine, leucine, lysine, phenylalanine and proline can be found as well as succinate. However, since the changes for these metabolites are partially contrary, they cannot serve as marker for discrimination of healthy and diseased mice between the two studies. Histidine, valine, tyrosine and acetate are only identified in study A, alanine, glutamine and methionine only in study B.

**Table 1:** Metabolites responsible for the differentiation between EBA mice and control mice

**Random forest**^**1**^				**PLS-DA**			
**Study A**		**Study B**		**Study A**		**Study B**	
**ppm**	**Metabolites**	**ppm**	**Metabolites**	**ppm**	**Metabolites**	**ppm**	**Metabolites**
0.97	leucine ↑^2^	1.07	n.a. ↑	8.35	n.a. ↑	8.43	n.a. ↑
3.17	histidine *↑	3.03	n.a. ↓	7.63	n.a. ↓	8.21	n.a.* ↑
1.01	isoleucine ↑	2.39	n.a. ↑	7.61	n.a. ↓	7.99	n.a.* ↑
1.05	valine ↑	1.21	n.a. ↑	7.51	n.a. ↓	7.91	n.a.* ↑
0.95	leucine, isoleucine ↑	3.07	n.a., tyrosine ↓	7.49	n.a. ↓	7.89	n.a.* ↑
2.33	n.a.^3^ ↓	0.91	n.a. ↑	7.45	phenylalanine ↑	7.75	tryptophane ↑
1.75	lysine, n.a. ↑	2.15	methionine, n.a. ↓	6.93	n.a., tyrosine ↑	7.73	tryptophane ↑
2.89	n.a.*	1.73	lysine ↓	6.91	tyrosine ↑	7.69	n.a. ↑
1.21	n.a. ↓	1.55	n.a. ↓	3.17	histidine* ↑	7.65	n.a. ↑
1.55	n.a. ↓	1.69	leucine ↓	2.89	n.a.* ↑	7.57	tryptophane, n.a. ↑
3.05	n.a, lysine. ↑	2.41	succinate ↑	1.57	n.a. ↓	7.55	tryptophane ↑
1.57	n.a. ↓	1.19	n.a. ↑	1.05	valine ↑	3.07	n.a. ↓
3.11	n.a. ↓	2.47	glutamine ↓	1.03	isoleucine, valine ↑	3.05	lysine, n.a. ↓
7.21	tyrosine ↑	3.05	n.a., lysine ↓	1.01	isoleucine ↑	3.03	n.a. ↓
2.41	succinate ↓	1.53	n.a. ↓	0.97	leucine ↑	1.21	n.a. ↑
1.03	isoleucine, valine ↑	3.01	n.a. ↓	0.95	leucine, isoleucine ↑		
2.27	valine ↑	2.65	methionine, n.a. ↓	0.81	n.a. ↑		
2.09	proline, n.a. ↓	7.33	phenylalanine ↑				
3.15	*	1.43	n.a., isoleucine ↓				
1.71	lysine, leucine ↑	1.47	alanine ↓				
3.19	n.a., histidine ↓	1.85	lysine, n.a. ↓				
1.93	acetate ↓	1.87	lysine ↓				
1.97	n.a.; arginine ↑	0.93	isoleucine ↑				
1.85	lysine, n.a. ↑	1.95	n.a.; arginine ↓				
2.51	n.a. ↓	1.51	n.a. ↓				
3.03	n.a.; lysine ↓	1.97	n.a.; arginine ↓				
1.59	n.a. ↓	2.43	glutamine, n.a. ↑				
7.19	tyrosine ↑	1.89	lysine ↓				
7.35	phenylalanine ↑	1.71	lysine, leucine ↓				
2.03	proline ↓	2.09	proline, n.a. ↓				

The ppm values of the buckets that are important in discriminating EBA and control mice are given with the metabolites that have been identified within these buckets. Buckets found in the random forest are listed according to the mean decrease of accuracy (see also Additional file 1: Figure S4).

^1^ buckets are in sequence according to their contribution to the discrimination of EBA mice and control mice.

^2^ arrows show increase or decrease of metabolite levels in EBA.

^3^ n.a. signals could not be assigned.

*strongly shifting peaks.

Collectively, an increase of isoleucine and phenylalanine, as well as a decrease of proline, can discriminate between EBA and control mice in both studies.

### Influence of batch effects on model building with PLS-DA

A PCA that includes spectra from both studies reveals a discrimination of EBA mice and control mice mostly in PCA component two (Additional file 1: Figure S3). Model building with PLS including both studies to predict the assignment of a serum sample to the group of EBA mice or control mice was not possible due to batch effects between both studies. Batch effects are well known e.g. in microarray experiments and the outcome of studies does not depend solely on biological variables, but also on variables like date or processing group of the samples [33]. The occurrence of batch effects could be caused by the age difference between mice in study A and study B at the time of blood sampling. Moreover, EBA mice in study B had symptoms over a longer time period, resulting in a different metabolic status of the mice in comparison to study A. A similar effect was observed by Martin *et al.* in their metabolite profiling of a mouse model of Crohn’s disease [17], where metabolites differed depending on age and duration of symptoms. Since the two diseased EBA groups show different metabolic patterns due to batch effects, future studies should be conducted with a larger number of mice to estimate the number of patients needed for a diagnostic setting for clinical use.

### Prediction of disease score from the data

In addition to discrimination of disease or healthy mice, metabolic profiles could be used for prediction of the disease activity score as a quantitative trait. For this purpose, data of EBA mice from studies A and B were combined and analyzed by PLS. The impact of the batch effect is not important for the PLS analysis since distribution of disease scores in both studies is actually the same (Additional file 1: Figure S1). Due to combination of both studies, the general validity of the PLS approach is improved.

**Figure 4:**
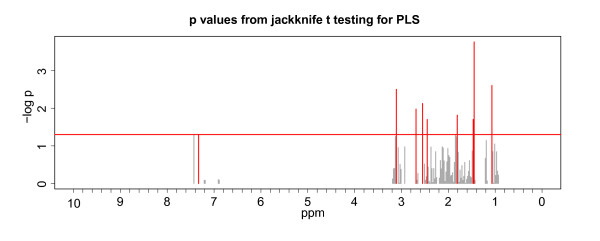
**Prediction of the EBA disease score.** P values from jackknife testing for PLS. A correlation of the disease score of EBA mice and control mice with the prediction of the score gives an r^2^ of 0.689. Relevant Buckets are colored red. For disease score prediction all diseased mice have been included. The vertical bar represents the significance threshold (−log 0.05). Number of components is 4.

To eliminate the risk of over-fitting the PLS model, a leave-one-out cross validation approach was used, i.e. each disease score to be predicted was left away from the data set used for PLS fitting. The resulting score prediction showed a high correlation with an r^2^ of 0.689 (Figure 4), confirming a good validity of the PLS approach.

The buckets that are relevant for this PLS model can be identified by a jackknife t test approach proposed by Martens and Martens [34]. We identified peaks of alanine, isoleucine, glutamine, lysine and phenylalanine. The buckets with these amino acids, except alanine, have a negative intensity in the coefficient scores plot (data not shown), i.e. negative intensity is pointing to a decrease in the disease score. The amino acids found by the PLS approach correspond to the amino acids found with the random forest approach.

### Metabolic differences point toward changes in energy balance

A random forest and PLS-DA approach was used to analyze the NMR data. It was demonstrated, that these algorithms provide reliable results when analyzing high dimensional NMR data [32]. We choose a 5% filter for the statistical analysis, meaning that peaks lower than 5% of the total peak intensity, are not considered in the calculation. The random forest model of this method gives the importance of the buckets for discrimination of EBA mice and control mice.

The observed metabolic changes indicate alterations in the energy metabolism in EBA mice versus control mice. An increased level of branched amino acids like leucine, isoleucine, valine in mice with EBA, as seen in study A, suggests a degradation of proteins to obtain energy.

Changes in the level of free amino acids were likewise found in previous studies characterizing the metabolic status during inflammation [35]. Alanine, as well as succinate, plays a role in energy metabolism as an intermediate in the citric acid cycle, also indicating changes in energy metabolism. Energy metabolism is also altered in IL-10 deficient mice, a mouse model of Crohn’s disease, due to decreased nutrient absorption through the intestine [17]. EBA is often associated with Crohn’s disease [36] and (at least in mice) induction of EBA is associated with decreased body-weight and a gastro-intestinal inflammatory response [37]. Glutamine is involved in energy metabolism [38] and the bucket intensity is decreased in study B according to the statistical analysis.

Proline and its derivative 4-hydroxyproline comprise about 25% of the total number of amino acids of collagen triple helices. The amino acid sequence glycine-proline-hydroxyproline is one of the most common motifs in collagen [39]. Little is known about the mechanism of antibody binding to COL7 or the impairment of its function. One may assume that COL7 is degraded as a result of autoantibody binding and/or the subsequently induced inflammatory response. This assumption is supported by the clinical observation that autoantibody deposition is not always detectable in lesional skin biopsies of patients with autoimmune blistering skin diseases [40,41]. The level of proline in the serum of EBA mice is decreased in comparison to the serum of control mice. The decrease of proline in the serum could be the consequence of an increased COL7 turnover. Glycine would allow testing this hypothesis, as it is the most abundant amino acid in collagen. However, it cannot be found with PLS analysis as it has just one peak lying in the exclusion area from 3.2 to 6.3 ppm.

The severity of inflammation can be associated with the methylation status of corresponding genes [42]. At the metabolic status an increase in methylation could be reflected by the lower level of methionine in study B. This has been observed before in the metabolic profiling of mucosal inflammation [43].

Immune responses during HIV infection or in ovarian carcinoma are accompanied by increased levels of phenylalanine [44,45]. Since we also observe increased levels of phenylalanine this reflects the immune response in EBA mice.

### Relevance to clinical applications

Blood samples are withdrawn in clinical routine and only few metabolites are usually determined. Metabolic profiles provide additional information on the patient’s status. In a metabolic study of rheumatoid arthritis patients the metabolic profiles changed during treatment thereby potentially allowing disease monitoring [19]. In our study metabolic profiles of EBA and control mice are different and the changes in metabolism correlate with the disease score in that experimental EBA mouse model. Future studies in patients, e.g. in a multi-centered setting, could therefore provide the basis to use the metabolic profiles to validate diagnosis, to identify potentially subgroups or co-morbidities of the patients. Additionally, identifying the cause of the metabolic changes will help to better understand disease mechanisms that are relevant in (adjuvant) therapy. It will be also of high interest if changes in metabolic profiles precede the onset of EBA as it was found for type I diabetes [46].

## Conclusion

The characterization of metabolites in experimental EBA by NMR spectroscopy allows discrimination of diseased from healthy mice. PLS-DA identified those metabolites that discriminate sera of mCOL7-immunized mice from sera of control mice. Changed metabolites are attributed to an increased energy demand, thus pointing towards a significant imprint of EBA on the metabolic profile. The information about metabolic changes due to EBA can be subsidiary for diagnosis but needs to be confirmed in a larger cohort that might be an obstacle due to the very low incidence of EBA. Altered metabolites refer to pathways that could be considered in developing therapeutic strategies for EBA. In addition, monitoring of alterations in the metabolic profile might provide a novel method to assess disease activity during treatment. Since blood samples are taken routinely, determination of metabolic profiles would deliver additional information on the patient’s status and could therefore be used to validate diagnosis or to identify potentially subgroups in the patients. Future investigations should also evaluate the specificity of the metabolic changes. It would be very interesting to know if other autoimmune skin blistering diseases show the same metabolic changes or if there are unique alterations for different diseases.

## Abbreviations

EBA: Epidermolysis bullosa acquisita; NMR: Nuclear magnetic resonance; PCA: Principal component analysis; PLS-DA: Partial least squares discrimination analysis; COL7: Type VII collagen.

## Competing interests

The authors state no competing interests.

## Authors’ contributions

SS and KS conducted the NMR experiments; SS, KS and AR analyzed the data, AR performed statistical analysis, MH executed the mouse experiments, RL and KS designed the study. All authors read and approved the final manuscript.

## Supplementary Material

Additional file 1 A box plot of the disease score (Figure S1); a PCA of EBA, GST immunized mice, not-immunized mice and mice treated with TiterMax® (Figure S2), a PCA of EBA and control mice of study A and B (Figure S3) and buckets that are found in the random forest according to the mean decrease of accuracy (Figure S4) are provided in the Additional file 1. Click here for file
