# Supplementary material for: Metabolite analysis distinguishes between mice with epidermolysis bullosa acquisita and healthy mice
Source: Orphanet J Rare Dis. 2013 Jun 26;8:93. doi: 10.1186/1750-1172-8-93 (PMC3703300; doi:10.1186/1750-1172-8-93)
Supplement: Additional file 1 — A box plot of the disease score (Figure S1); a PCA of EBA, GST immunized mice, not-immunized mice and mice treated with TiterMax® (Figure S2), a PCA of EBA and control mice of study A and B (Figure S3) and buckets that are found in the random forest according to the mean decrease of accuracy (Figure S4) are provided in the Additional file 1. [file 1750-1172-8-93-S1.pdf]

Supplemental information

Metabolite analysis distinguishes between mice with epidermolysis bullosa acquisita and healthy mice

Sarah Schönig[1,2], Andreas Recke[1,3], Misa Hirose[1,3], Ralf J. Ludwig[1,3] and Karsten Seeger[1,2]

[1] Excellence Cluster Inflammation at Interfaces, Schleswig-Holstein, Germany

[2] Department of Chemistry, University of Lübeck, Lübeck, Germany

[3] Department of Dermatology, University of Lübeck, Lübeck, Germany

Correspondence: Karsten Seeger, Department of Chemistry, University of Lübeck, Ratzeburger Allee 160, 23562 Lübeck, Germany, E-mail: karsten.seeger@chemie.uni-luebeck.de

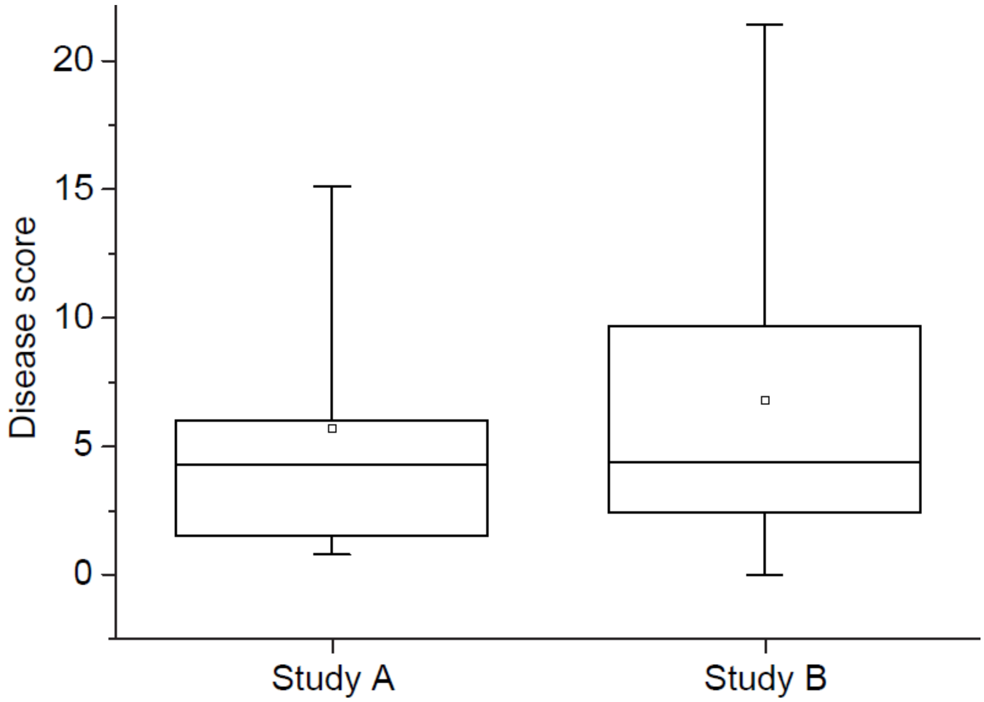

Supplemental figure 1 Box plot of the disease score. The disease score is expressed as the percentage of body surface area affected by lesions of the EBA mice in study A and study B. The box represents median, 25th and 75th percentile. Whiskers represent the 1.5 interquartile range. Mean is represented by a rectangle. The disease score distribution can be regarded as equal according a Wilcoxon rank sum test (p>>0.2).

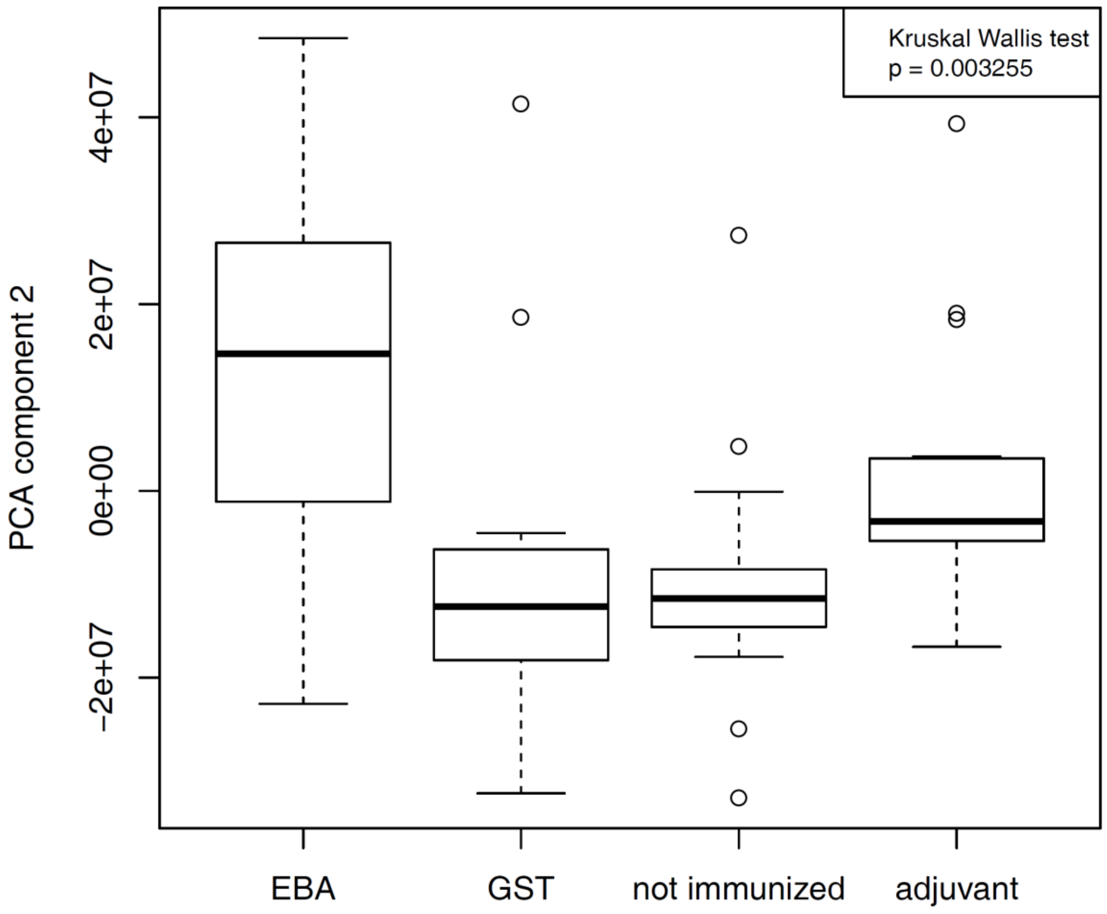

Supplemental figure 2 Complete representation of control groups used in study B. Box & whisker plots of principal component 2. PCA shows separation of EBA mice (n=15) from control groups (n=15 each) along component 2. Within Study B sera of not immunized mice and mice injected only the adjuvant TiterMax® had been included. However, since the not immunized mice and the adjuvant group were not treated with an antigen and therefore did not developed an immune response, these two groups are considered as somehow improper controls for analyzing metabolic changes due to EBA. Outliers represented as circles.
A PCA of only EBA and GST controls is shown in figure 2 of the manuscript.

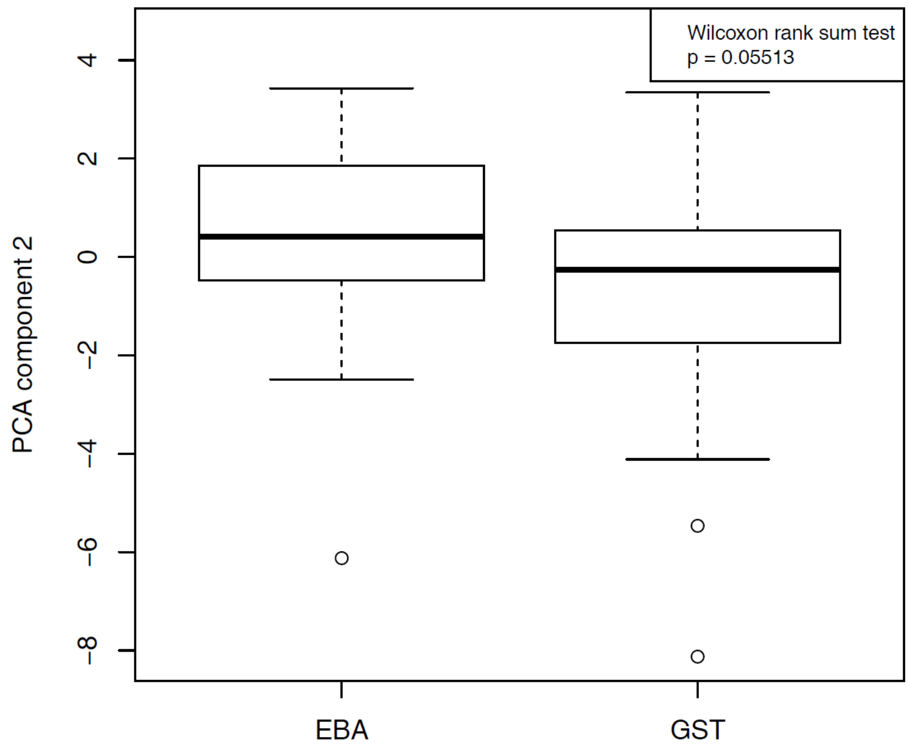

Supplemental figure 3 Box & whisker plot of PCA component 2 of combined studies A and B shows discrimination of EBA versus control mice. The boxplots of the robust PCA are standardized with outliers represented as circles.

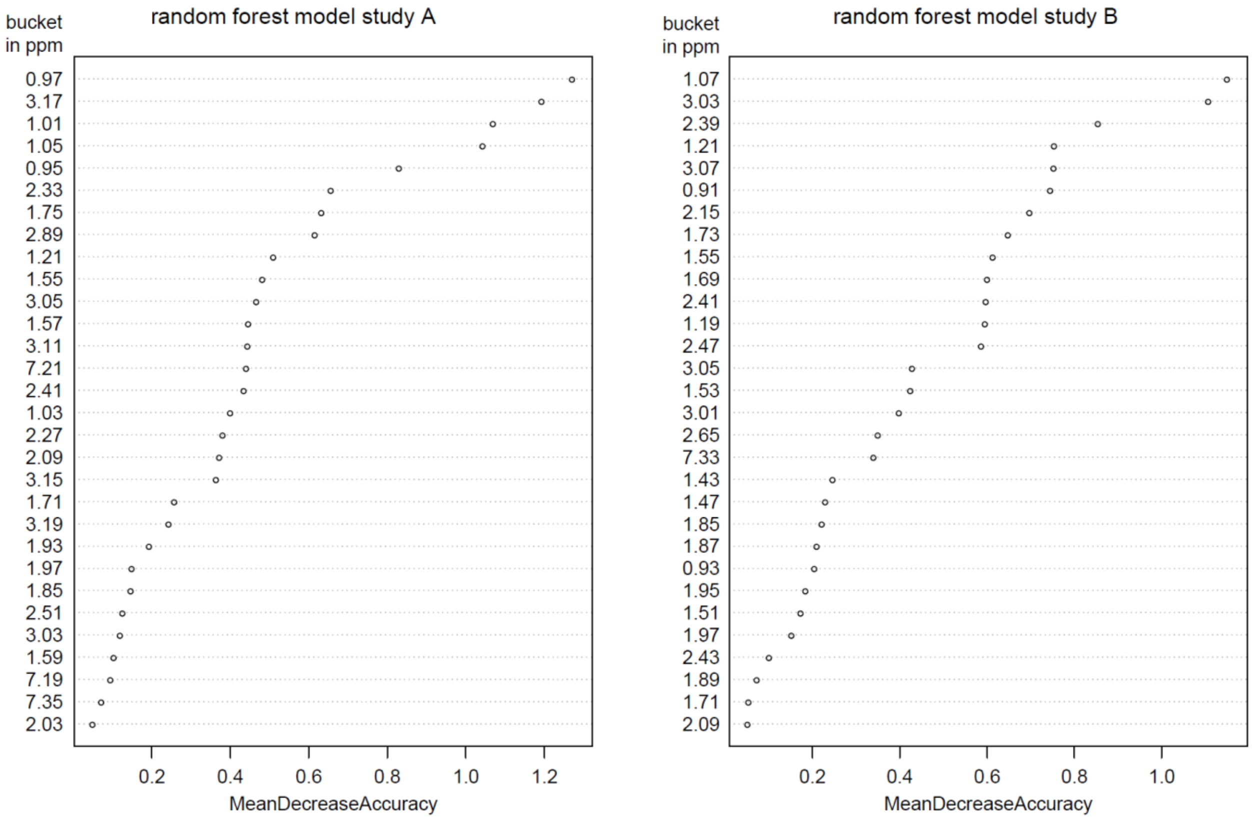

Supplemental figure 4 Buckets identified in the random forest are plotted according to the mean decrease in accuracy.
